# Object tracking algorithm based on deformable attention mechanism

**DOI:** 10.1038/s41598-026-43147-x

**Published:** 2026-03-06

**Authors:** Qiaoling Liu, Na Yu, Jinfu Cheng

**Affiliations:** 1 School of Electronic Information and Electrical Engineering, Chengdu University, Chengdu, 610106 China; 2 Entrepreneurship College, Chengdu University, Chengdu, 610106 China; 3 Department Engineering, Datong Vocational and Technical College of Coal, Datong, 037000 China

**Keywords:** Object tracking algorithm, Deformable attention mechanism, ResNet-18, Bidirectional feature pyramid network, Kalman filter, Engineering, Mathematics and computing

## Abstract

Occlusion, sudden illumination changes, and rapid motion in complex scenes severely degrade the robustness of existing object tracking methods. To address this issue, this paper proposes a novel object tracking algorithm that integrates a deformable attention mechanism. The method first embeds a deformable attention module into the ResNet-18 feature extraction network to enable adaptive enhancement of target key features. Second, the method adopts an improved Bidirectional Feature Pyramid Network as the feature fusion module to enhance the representational capability of multi-scale features. Finally, the method incorporates a dynamic Kalman filtering prediction module to improve the algorithm’s adaptability to changes in the target’s motion state and its continuous tracking capability. Experimental results show that the improved feature extraction network achieves an average overlap rate and success rate of 61.5% and 68.4%, respectively, on the GOT-10k dataset, with a computational load of only 1.96 GFLOPs and an increase of only 0.23 M in parameters. On the MOT20 dataset, the proposed object tracking network achieves a Multiple Object Tracking Accuracy of 77.5%, an Identity F1 Score of 77.0%, with 54.6% Majority of Tracked Trajectories and 12.5% Majority of Lost Trajectories. Its tracking performance surpasses that of the compared object tracking algorithms. These results confirm the efficacy of the Deformable Attention Mechanism and present a robust solution for complex dynamic tracking scenarios.

## Introduction

Target tracking technology, as a significant research direction in computer vision, plays a pivotal role in practical applications such as intelligent surveillance, autonomous driving, and smart retail^1^. In recent years, with the rapid advancement of deep learning techniques, deep learning-based target tracking methods have demonstrated remarkable advantages^2^. Compared to traditional approaches, these methods can automatically extract more discriminative features and employ direct, effective tracking strategies, achieving reliable and efficient tracking performance^3^. However, object tracking algorithms still face multifaceted challenges. First, maintaining high accuracy under complex environmental disturbances requires further investigation^4,5^. Second, issues such as feature drift and matching errors caused by occlusions and lighting variations demand urgent solutions^6^. Although existing research has partially addressed these problems by incorporating motion estimation and attention mechanisms, a unified solution remains elusive^7^. Notably, the Deformable Attention Mechanism (DAM) addresses geometric variations by learning dynamic sampling offsets to focus on critical regions, showing promise in tasks like object detection. However, systematically integrating DAM’s geometric adaptability into tracking frameworks and achieving closed-loop collaboration with modules like motion estimation remains an unexplored challenge.

To address this, this study innovatively designs a target tracking algorithm that integrates the Deformable Attention Mechanism. Its core innovation lies in breaking the paradigm of independent optimization across modules in traditional tracking models, constructing a closed-loop system where feature extraction, feature fusion, and state estimation are globally coordinated by detection reliability. First, a deformable attention mechanism is embedded within the ResNet-18 backbone network, enabling dynamic adjustment of the receptive field according to target confidence to achieve adaptive focusing on key features. Subsequently, a DAM-optimized weighted Bidirectional Feature Pyramid Network (BiFPN) is designed, which efficiently fuses multi-scale discriminative features through bidirectional cross-scale fusion and quadratic attention. Finally, the study integrates a dynamic Kalman filter mechanism to intelligently select tracking modes based on detection confidence, enabling continuous and accurate target tracking. The proposed improvements are expected to enhance tracking accuracy and robustness, providing a technical paradigm for continuous, precise tracking in complex environments. This offers reliable technical support for practical applications such as intelligent video surveillance and automated driving.

## Related works

Researchers have proposed improvement strategies at multiple levels to address object tracking challenges in complex scenes. Wang Y et al. built a new tracking framework based on the YOLOv8 detector. By specifically optimizing the IoU matching and loss function in the ByteTrack tracking algorithm, they achieved MOTA scores of 74.0% and 66.8% on the MOT17 and MOT20 datasets, respectively, significantly enhancing ByteTrack’s tracking robustness and performance in complex scenarios^8^. To address the challenges of infrared target detection and tracking in dense urban environments, Zha et al. integrated multiple image enhancement techniques, adopted MobileViTv3 to refine the backbone network, and designed specialized infrared feature extraction and efficient matching modules. These innovations significantly enhanced detection accuracy, speed, and overall multi-target tracking performance in low-contrast, complex settings^9^. Addressing background interference challenges in shallow-water biological detection and tracking, Liu Y et al. introduced an attention mechanism into the YOLOv5 feature extraction network to suppress complex background noise, achieving a 3.2% improvement in average detection accuracy. They further combined this enhanced detector with a cascaded matching strategy, effectively reducing target identity switching during prolonged occlusions^10^. Regarding network architecture, Nguyen T T et al. proposed an end-to-end multi-camera multi-object tracking solution based on Transformers and graph neural networks. This model effectively addresses the challenges of sparse annotated data and cross-camera rule adaptation in practical applications through three major modules: language model detection, graph association, and text embedding generation^11^. Addressing multi-vehicle tracking demands in intelligent transportation scenarios, Ishtiaq N’s research team proposed an enhanced stochastic finite-set filtering framework. This innovative approach incorporates modeling of target interactions to construct a multi-object tracking algorithm with interaction perception capabilities, with its effectiveness validated through experiments^12^. Regarding computational efficiency optimization, Péter Szántó and colleagues introduced field-programmable gate array (FPGA) technology. They accelerated feature extraction by employing lightweight feature extraction networks and optimized computational efficiency during matrix operations through fixed-point arithmetic, significantly enhancing overall processing speed^13^. Razak R N et al. combined deep learning with simple online real-time tracking algorithms to propose a frame-discarding optimization method. By dynamically adjusting the number of processed video frames, this approach effectively reduced computational load while maintaining tracking accuracy. Experimental results demonstrated that this strategy not only shortened algorithm runtime but also significantly improved overall performance^14^. Addressing target loss in complex scenes, Alamri F S’s team discretized continuous intervals into subintervals and employed probability distributions for filtering. By eliminating low-probability regions to focus the search scope, they validated the model’s effectiveness and practicality in real-world scenarios^15^.

Attention mechanisms provide an effective pathway to enhance model discriminative power by focusing on key information. Among these, DAM learns dynamic sampling offsets to adaptively focus on geometrically critical regions of targets, offering novel insights for handling deformation and occlusion. Ayman B et al. introduced a novel deep attention module by incorporating depth-related adaptive thresholds and influence factors, optimizing depth information utilization in both geometric and semantic modules when applying DAM attention to simultaneous localization and mapping tasks^16^. Ge Q’s research team proposed an enhanced real-time detection algorithm for metal bipolar plate defect detection. To concentrate the algorithm on critical feature regions, they introduced a deformable DAM attention mechanism, experimentally validating its effectiveness^17^. However, research remains insufficient on systematically integrating DAM’s geometric adaptability into multi-object tracking frameworks and achieving closed-loop collaboration with modules like motion estimation.

Furthermore, the evolution of benchmark datasets in target tracking poses increasingly stringent challenges to tracking algorithms. MOT20 has become a litmus test for evaluating an algorithm’s generalization capability and dense occlusion handling by introducing extremely crowded scenes. DanceTrack, on the other hand, specifically assesses an algorithm’s core capabilities in motion modeling and spatio-temporal association using highly similar-looking dancers with complex movements, rather than relying on appearance discriminative power. The emergence of these datasets highlights the severe challenges current tracking algorithms still face in complex motion modeling and generalization in high-density scenes.

In summary, existing research has made significant progress in detector design, network architecture optimization, and computational efficiency enhancement. These efforts have improved the performance and practicality of tracking systems from various perspectives by introducing attention mechanisms, designing end-to-end architectures, and implementing lightweight strategies. However, most existing improvements focus on independent optimization of individual modules, lacking a systematic design for the collaborative mechanisms among feature extraction, multi-scale fusion, and motion estimation. Additionally, existing methods exhibit insufficient adaptability and robustness when handling target deformation, severe occlusion, and appearance similarity interference, particularly lacking a global mechanism that dynamically coordinates modules based on scene confidence. Based on this analysis, this study proposes a systematic solution. By integrating the DAM attention mechanism into the ResNet-18 backbone network and the BiFPN feature fusion network, and designing a dynamic Kalman filter module guided by detection confidence, a globally coordinated tracking framework is constructed. This framework aims to achieve deep synergy among feature extraction, multi-scale fusion, and behavior prediction, directly addressing the shortcomings of existing methods in module coordination and scene adaptability to enhance tracking performance in complex dynamic scenarios.

To clearly illustrate the key distinctions between existing methods and the proposed approach, Table 1 provides a systematic comparison in terms of research methodology, core technology, advantages, and limitations. The table is presented below.

**Table 1:** Literature comparison analysis table.

Method	Core approach	Main optimization	Limitations	Ref
YOLOv8-ByteTrack optim.	Detector-tracker co-optimization	Improved robustness on MOT17/20	Weak module synergy; limited geometric adaptation	^8^
Attn-enhanced deepSORT	Attention-based detector + cascade matching	Reduced ID switches under occlusion	Tracking performance heavily detector-dependent	^9,10^
Transformer-GNN MOT	End-to-end multi-camera tracking	Handles label scarcity & cross-camera association	High computational cost; generalization in crowds unverified	^11^
Interaction-aware RFS filter	Motion model with interaction modeling	Better tracking in structured traffic scenes	Scene-specific; poor generalization	^12^
Efficiency optimization	Hardware/algorithmic speed-up	Significant runtime reduction	Potential accuracy trade-off; does not address core tracking challenges	^13^ ^14^
Probabilistic re-search	Post-hoc target re-identification	Efficient recovery of lost targets	A reactive module, not integrated into main tracking loop	^15^
DAM-Track	Global confidence-coordinated closed-loop system	Systematic synergy; geometric adaptation to deformation/occlusion	Framework complexity requires careful tuning	This work

As shown in Table 1, existing work primarily focuses on isolated improvements in individual stages such as detection, association, or computational efficiency, generally lacking systematic coordination and geometric adaptability. In contrast, this study achieves systematic optimization of feature extraction, fusion, and motion estimation through confidence-based coordination and the DAM attention mechanism.

## Methods

### Optimization design of feature extraction network with DAM integration

**Fig. 1:**
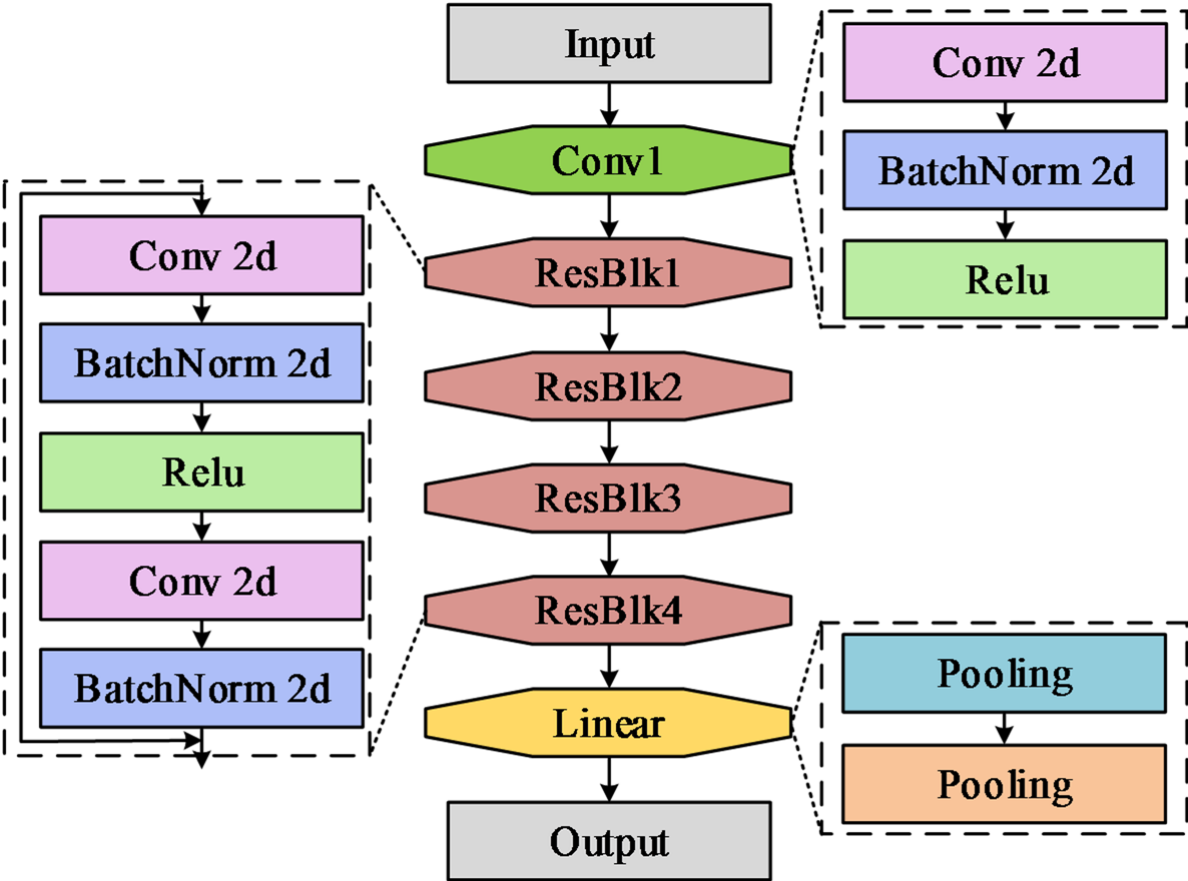
Architecture of the ResNet-18 feature extraction network.

The object tracking algorithm first takes a video sequence as input. After initializing the tracking target, the algorithm accurately estimates target movement in subsequent frames. In object tracking algorithms, filtering-based tracking frameworks and Siamese tracking frameworks receive most of the attention from researchers and become mainstream models in the current tracking field^18,19^. However, traditional filtering-based frameworks lose the target easily when scale variation or continuous occlusion occurs^20^. Siamese tracking frameworks, due to their local linear matching process, often fall into local optima^21^. To address these challenges, this paper employs ResNet-18 as the feature extraction network. The advantages of ResNet-18 lie in its ability to preserve the integrity of deep features through residual connections, making it adaptable to long-term variations in target appearance. Additionally, its concise residual block design significantly reduces computational complexity while maintaining discriminative power, thus meeting real-time tracking requirements^22,23^. Based on this, the study constructs a feature extraction network architecture with ResNet-18 as the backbone, as illustrated in Fig. 1.

As shown in Fig. 1, ResNet-18 adopts the classic residual network architecture, comprising an initial convolutional layer, four residual module stages, and a final global pooling and fully connected layer^24^. Each stage of the network consists of several stacked residual blocks, with each residual block composed of two 3 × 3 convolutional layers and implementing identity mapping through shortcut connections^25,26^. For a basic residual block, its mathematical expression is given by Eq. (1).

1 $$\left\{ \begin{gathered} y=F\left( {x,\left\{ {{W_i}} \right\}} \right)+x \hfill \\ {x_{l=1}}=f\left( {{y_l}} \right) \hfill \\ \end{gathered} \right.$$

In Eq. (1), *x* represents the input feature, *F *denotes the residual function (typically composed of several convolutional layers), ${W_i}$ stands for the weight parameters, and *f* is the activation function. For deep networks, the mathematical expression of a residual block is given by Eq. (2).

2 $${x_{l=1}}=x+F\left( {{x_l},{W_l}} \right)$$

**Fig. 2:**
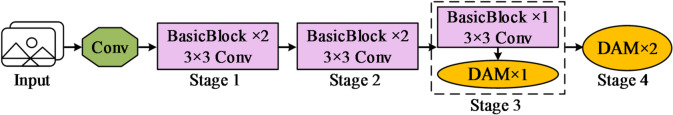
Flowchart of feature extraction with the DAM incorporated.

ResNet-18 provides an effective feature representation foundation for object tracking due to its residual structure and moderate computational complexity, However, in multi-object tracking scenarios, common challenges such as non-rigid deformation, abrupt pose changes, and partial occlusion among targets arise. The fixed receptive field in ResNet-18’s standard convolutional operations cannot dynamically adapt to such displacements. This often leads to the extraction of interfering features from backgrounds or adjacent targets in deformed or occluded regions, limiting its accuracy in representing dynamic target features. To address this, the study introduces the DAM attention mechanism. Building upon the fixed weighted summation of standard convolutions, this mechanism incorporates continuous spatial offsets predicted by a lightweight quantized network, enabling the receptive field to adaptively deform with content. This design allows the model to precisely focus on key semantic regions of moving targets through continuous modeling of local deformations, enhancing robustness against deformation and occlusion. Simultaneously, the offset generation process in DAM optimizes tracking performance, enabling the network to implicitly acquire prior knowledge of the spatial distribution of discriminative features. This achieves alignment between feature selection and task objectives. Furthermore, its sparse sampling strategy delivers these performance improvements while maintaining computational efficiency comparable to standard convolutions. The feature extraction process incorporating DAM is given in Fig. 2.

As shown in Fig. 2, the specific implementation of embedding DAM within ResNet-18 involves first inserting a single DAM module in the third stage. This enables the network to dynamically adjust its receptive field based on the target structure, thereby capturing preliminary geometric deformation information. Subsequently, all standard 3 × 3 convolutions in the fourth stage are replaced with DAM attention modules, further enabling adaptive aggregation of global context at deep semantic levels. The first two stages retain standard convolutional structures, focusing on extracting fundamental spatial features. Compared to DAM, traditional self-attention mechanisms capture complex relationships between elements by distributing weights within sequences, as illustrated by its core calculation formula in Eq. (3).

3 $$Attention\left( {Q,K,V} \right)=soft\hbox{max} \left( {\frac{{Q{K^T}}}{{\sqrt {{d_k}} }}} \right)V$$

In Eq. (3), $\sqrt {{d_k}}$ represents the scaling factor. The core advantage of DAM lies in its ability to overcome limitations imposed by feature map dimensions by dynamically predicting sampling offsets, thereby confining computations to locally critical regions. Suppose the input feature map is $x \in {R^{C \times H \times W}}$, and the reference point is *P*. The DAM calculation is shown in Eq. (4).

4 $$DeforAttn\left( {Q,P,X} \right)=\sum\limits_{{i=1}}^{{{N_{head}}}} {{W_i}} \sum\limits_{{j=1}}^{{{N_{hey}}}} {{A_{ij}}} \cdot W_{i}^{\prime } \cdot X\left( {P+\Delta {P_{ij}}} \right)$$

**Fig. 3:**
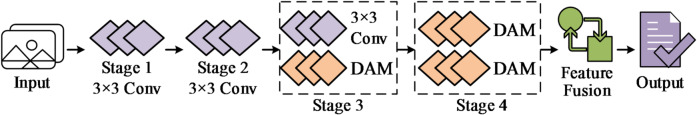
Flowchart of the ResNet-18-DAM feature extraction network.

In Eq. (4), ${N_{head}}$ is the number of attention heads, *i* and *j* represent the indices of attention heads and sampling points, *N* represents the number of sampling points per attention head, ${A_{ij}}$ represents the attention weights, *W* represents the learnable weights, $\Delta {P_{ij}} \in {R^2}$ represents the offset of *P*, and $X\left( {P+\Delta {P_{ij}}} \right)$ represents the feature at position $P+\Delta {P_{ij}}$. This design transforms DAM from global dense computation to local sparse computation, significantly reducing computational complexity while enhancing the ability to extract key features of geometric deformation. The research integrates DAM into the ResNet-18 network, forming a ResNet-18-Dynamic Attention Mechanism (ResNet-18-DAM) feature extraction network based on a deformable attention mechanism. Its workflow is illustrated in Fig. 3.

As shown in Fig. 3, the first two stages of the ResNet-18-DAM feature extraction network employ standard convolution kernels to extract basic visual features, maintaining geometric stability. Stage three adopts a hybrid structure: standard convolution first preserves feature consistency, followed by DAM. Through two parallel lightweight convolutional branches, the DAM module dynamically generates spatial offsets and attention masks, thereby achieving preliminary geometric adaptive learning. Stage four fully utilizes the DAM attention mechanism, employing a two-level cascaded module for in-depth offset learning and adaptive feature aggregation, enabling it to specifically address complex deformations and occlusions. Ultimately, the network outputs multi-scale adaptive features that provide semantically discriminative representations for the subsequent tracking head.

### Design and optimization of object tracking algorithm with DAM integration

The object tracking system is structured around three key modules: feature extraction, feature fusion, and predicting target positions. Based on the improved ResNet-18-DAM feature extraction network, this study further optimizes the feature fusion module and the target prediction module to construct a complete object detection algorithm. Among existing feature fusion networks, BiFPN stands out due to its cross-scale bidirectional fusion mechanism. Compared with traditional algorithms, BiFPN combines multi-level features efficiently through weighted bidirectional connections, retaining low-level detail features while fusing high-level semantic features^27^. Therefore, this paper employs BiFPN as the backbone of the feature fusion module. BiFPN achieves more efficient feature combination by employing bidirectional cross-scale connections along with weighted feature aggregation. Weighted feature fusion merges features at each level and performs a weighted sum with learnable weights, as shown in Eq. (5)^28^.

5 $$O=\sum\limits_{{\overset{\lower0.5em\hbox{$\smash{\scriptscriptstyle\frown}$}}{i} }} {\frac{{{\omega _{\overset{\lower0.5em\hbox{$\smash{\scriptscriptstyle\frown}$}}{i} }}}}{{\varepsilon +\sum\nolimits_{{\overset{\lower0.5em\hbox{$\smash{\scriptscriptstyle\frown}$}}{j} }} {{\omega _{\overset{\lower0.5em\hbox{$\smash{\scriptscriptstyle\frown}$}}{j} }}} }}} \cdot {F_{\overset{\lower0.5em\hbox{$\smash{\scriptscriptstyle\frown}$}}{i} }}$$

In Eq. (5), *O* represents the fused output feature, $${F_{\overset{\lower0.5em\hbox{$\smash{\scriptscriptstyle\frown}$}}{i} }}$$ represents the $$\overset{\lower0.5em\hbox{$\smash{\scriptscriptstyle\frown}$}}{i}$$-th input feature from different levels, $${\omega _{\overset{\lower0.5em\hbox{$\smash{\scriptscriptstyle\frown}$}}{i} }}$$ represents the learnable weights, and $$\varepsilon$$ represents a small value. Taking a three-level pyramid as an example, the expression for top-down fusion in the BiFPN bidirectional fusion process is shown in Eq. (6).

6 $$\bar {P}_{4}^{{td}}=Conv\left( {\frac{{{\omega _1}{{\bar {P}}_5}+{\omega _2}\operatorname{Re} size\left( {\bar {P}} \right)}}{{{\omega _1}+{\omega _2}+\varepsilon }}} \right)$$

In Eq. (6), $\bar {P}$ represents the feature level. The bottom-up fusion expression is shown in Eq. (7).

7 $$\bar {P}_{4}^{{out}}=Conv\left( {\frac{{{\omega _1}{{\bar {P}}_4}+{\omega _2}\bar {P}_{4}^{{td}}+{\omega _3}\operatorname{Re} size\left( {{{\bar {P}}_3}} \right)}}{{{\omega _1}+{\omega _2}+{\omega _3}+\varepsilon }}} \right)$$

**Fig. 4:**
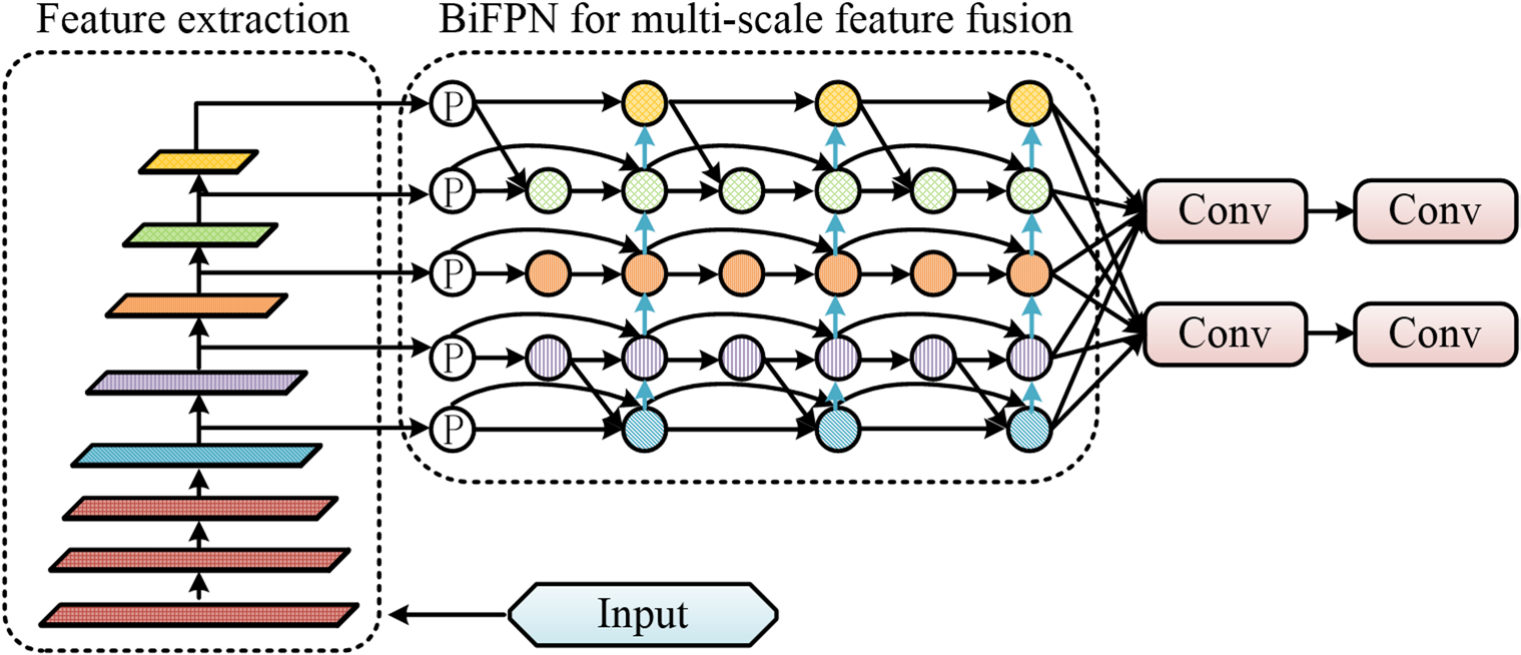
Target feature fusion process using BiFPN.

The process of using BiFPN for target feature fusion is shown in Fig. 4.

As shown in Fig. 4, the feature fusion process of BiFPN begins with initialization, extracting multi-scale features from the backbone network. This is followed by the first round of top-down and bottom-up fusion, culminating in iterative bidirectional fusion. Multiple BiFPN layers are stacked according to task requirements. Although BiFPN effectively fuses multi-scale features through bidirectional weighted fusion, its fixed receptive field struggles to adapt to cross-layer feature misalignment caused by target occlusion, deformation, and dense interactions in MOT. This misalignment leads to cross-contamination of features from different targets during fusion, increasing the risk of identity switching^29^. DAM, however, precisely focuses on key target regions through dynamic prediction of sampling offsets and adaptive weights. Therefore, to retain BiFPN’s multiscale feature fusion advantages while endowing the network with dynamic spatial attention adjustment capabilities, this study deeply integrates DAM into each bidirectional fusion node. The BiFPN output feature is denoted as $\bar {P}_{l}^{{out}}$. DAM predicts sampling offsets and weight masks for each position, as shown in Eq. (8).

8 $${p_{Sampling}}=p+\Delta p$$

**Fig. 5:**
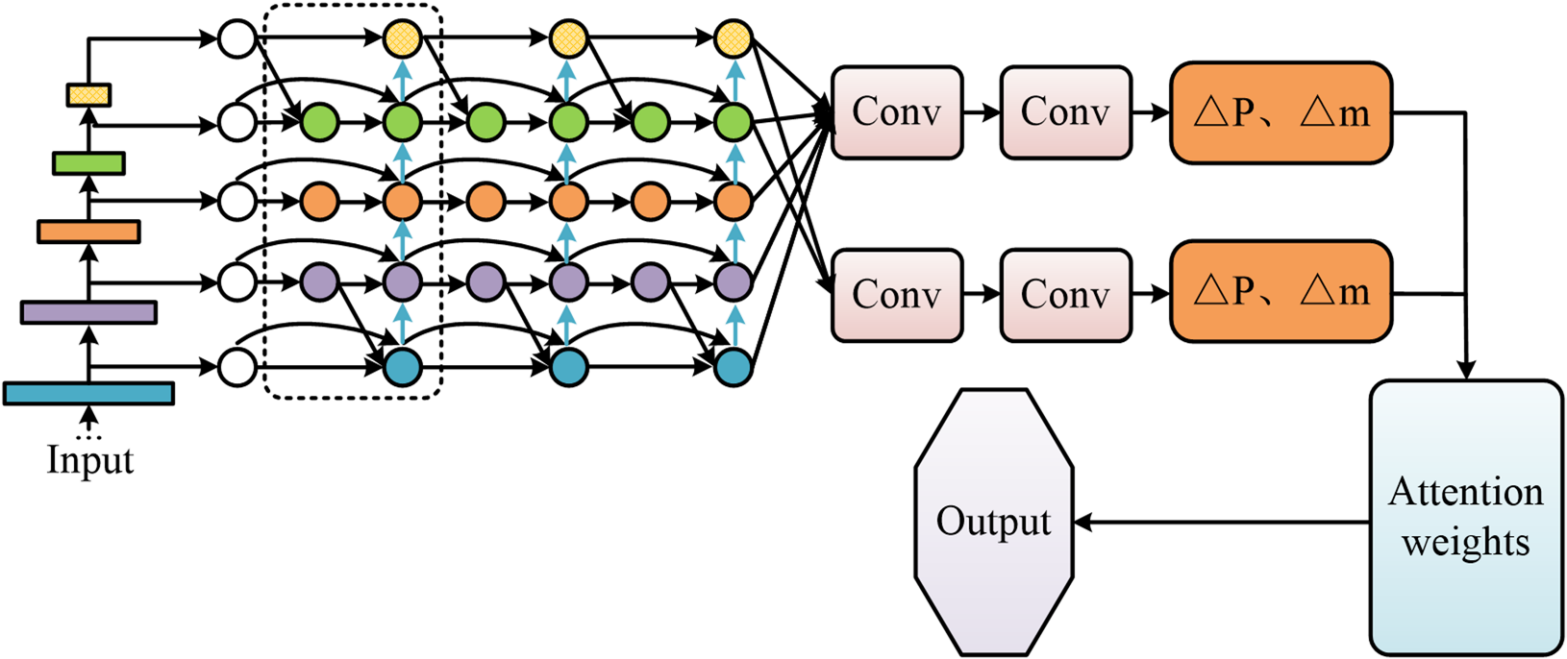
DAM-optimized BiFPN feature fusion network process.

In Eq. (8), $\Delta p0$ represents the learned offsets. Unlike traditional BiFPN, which relies solely on fixed-weight linear fusion, the specific operation involves dynamically generating a set of spatial offset fields and attention weight masks using the DAM module based on the contextual information of the current input features before performing feature aggregation at each node. This offset field performs deformable resampling on features from adjacent layers, enabling geometric adaptive alignment across feature scales. Subsequently, the attention mask applies importance-weighted fusion to the aligned features, allowing the network to autonomously focus on key regions and discriminative parts of the target. Simultaneously, DAM performs deformable sampling on feature maps, directing the BiFPN network’s focus toward critical regions. The BiFPN feature fusion network workflow optimized with DAM is illustrated in Fig. 5.

As shown in Fig. 5, the DAM-optimized BiFPN feature fusion network first extracts multi-scale features from the backbone network and inputs them into BiFPN for bidirectional cross-scale fusion. Bidirectional cross-scale fusion includes top-down and bottom-up paths. The top-down path samples high-level semantic features and fuses them with low-level features. The bottom-up path restores detail information through down-sampling to form an initial multi-scale feature fusion. These enhanced features will be utilized for subsequent object detection and appearance feature extraction. However, maintaining stable object identity across consecutive frames requires not only accurate appearance representation but also reliable motion prediction. Therefore, this study optimizes the motion prediction module within the tracking framework. In the field of tracking, Kalman filtering has become a classic choice for prediction modules due to its efficient linear estimation capabilities^30^. Consequently, Kalman filtering is selected as the backbone network for the prediction module within the object detection algorithm. However, in multi-object tracking, traditional Kalman filtering operates under the linear assumption of uniform object motion, focusing on state estimation with observations as corrections. This approach accumulates errors over time and leads to trajectory drift during occlusions^31^. With the significant improvement in modern detector accuracy, high-confidence detection boxes themselves possess high reliability and can serve as the dominant information source for motion estimation. To address this, this study proposes a dynamic Kalman filtering method that adopts an observation-centered strategy, replacing the estimation-centered approach of traditional Kalman filtering. Specifically, the study introduces a confidence modulation function $\lambda \left( {{c_t}} \right)$based on the traditional Kalman filter update equation., which is linked to the observation noise covariance, i.e., by setting ${R^{ - 1}} \propto \lambda \left( {{c_t}} \right)$. This leads to the derivation of the dynamic Kalman gain, and its mathematical expression is given in Eq. (9).

9 $$K_{t}^{{dyn}}={\operatorname{P} _{t\left| {t - 1} \right.}}{H^T}{\left( {H{\operatorname{P} _{t\left| {t - 1} \right.}}{H^T}+\lambda {{\left( {{c_t}} \right)}^{ - 1}}R} \right)^{ - 1}}$$

**Fig. 6:**
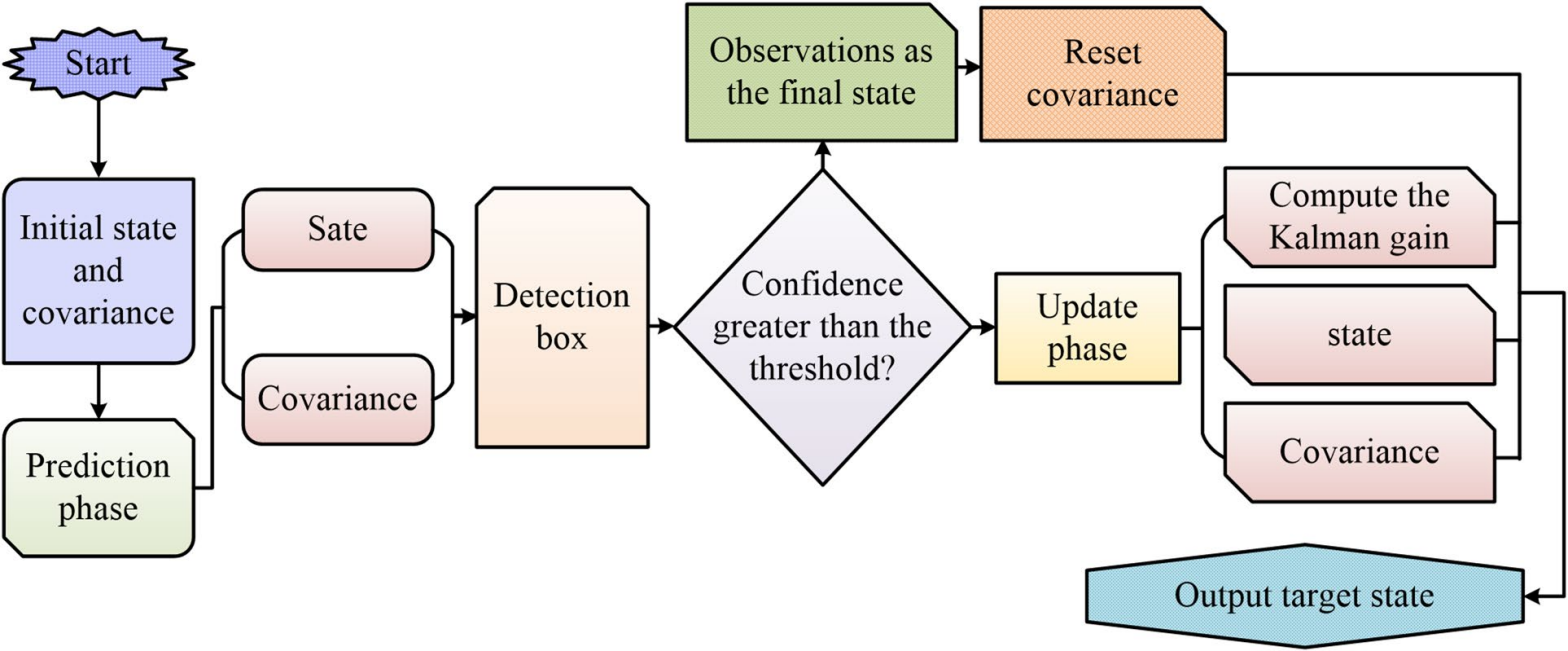
Target tracking prediction process based on dynamic Kalman filter.

In Eq. (9), the parameter ${c_t}$ denotes the detection confidence of the current frame. The modulation function $\lambda \left( {{c_t}} \right)$is defined as follows: when the detection confidence $\lambda \left( {{c_t}} \right)$ exceeds the preset threshold T, the function takes the value 1; otherwise, its value decays linearly according to the ratio of ${c_t}$ to *T*. This design enables the system to fully trust detection results under high confidence observations, directly interrupting error propagation. Under low confidence conditions, the filter’s correction strength is smoothly adjusted based on observation quality. The threshold *T* is selected according to the observation reliability principle. Analysis of target detector output distribution on the validation set establishes the 90th percentile of confidence distribution as $T=0.9$. This value effectively filters out high-reliability detection samples while avoiding frequent error accumulation caused by excessively high thresholds.The process of target tracking prediction using dynamic Kalman filtering is shown in Fig. 6.

As shown in Fig. 6, the dynamic Kalman filter makes intelligent decisions based on the detector confidence threshold. When the confidence is higher than the threshold, the detection result is directly used as the tracking box and the target ID is updated. Otherwise, the detection box and the prediction box are fused as the final prediction result. The traditional Kalman filter mainly includes the prediction phase and the update phase. In the prediction phase, the state equation predicts the next frame position, as shown in Eq. (10).

10 $${\hat {x}_t}=F \cdot {x_{t - 1}}+\upsilon$$

In Eq. (10), ${\hat {x}_t}$ is the state estimate, *F* is the state transition matrix, and $\upsilon$is the process noise. The covariance prediction equation is shown in Eq. (11).

11 $$P_{k}^{ - }={F_k}{P_{k - 1}}F_{k}^{T}+{Q_k}$$

In Eq. (11), $P_{k}^{ - }$ represents the prior estimation covariance, ${P_{k - 1}}$ represents the posterior covariance of the previous time, and ${Q_k}$ represents the process noise covariance. In the update phase, the optimal estimate is obtained by fusing the current posterior information with the estimated state. The Kalman gain in the update phase is calculated as shown in Eq. (12).

12 $${K_k}=P_{k}^{ - }H_{k}^{T}{\left( {H_{k}^{{}}P_{k}^{ - }H_{k}^{T}+{R_k}} \right)^{ - 1}}$$

In Eq. (12), ${K_k}$ represents the Kalman gain, which is the weight for balancing prediction and measurement. The state update equation is shown in Eq. (13).

13 $${\hat {x}_k}=\hat {x}_{k}^{ - }+{K_k}\left( {{z_k} - H_{k}^{{}}\hat {x}_{k}^{ - }} \right)$$

**Fig. 7:**
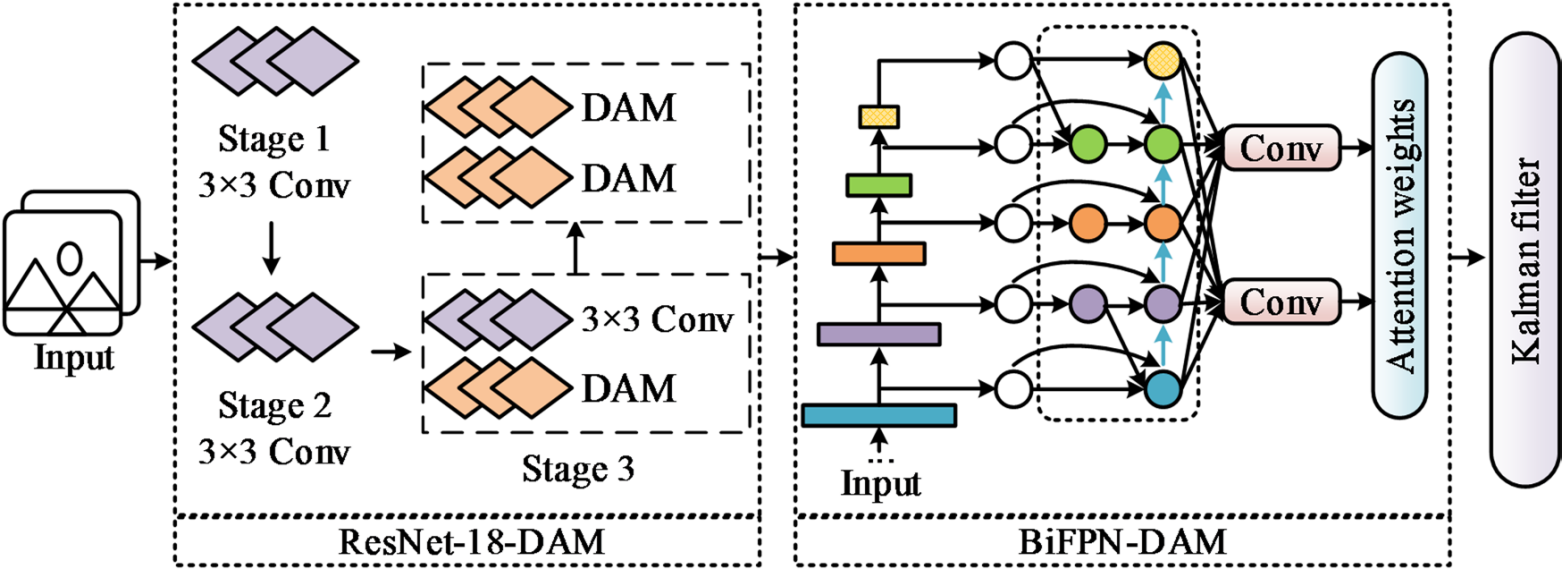
DAM-based object tracking algorithm process.

In Eq. (13), $\hat {x}_{k}^{ - }$ represents the optimal posterior estimate at time *k*, ${z_k}$ represents the actual observation value, and $\left( {{z_k} - H_{k}^{{}}\hat {x}_{k}^{ - }} \right)$ represents the observation residual. In summary, this study proposes a Deformable Attention Mechanism-Based Object Tracking Algorithm (DAM-Track), featuring improvements across three core components. For the backbone network, ResNet-18-DAM is constructed by replacing specific convolutional layers in Stages 3 and 4 of ResNet-18 with DAM modules, endowing it with a dynamic receptive field. For feature fusion, a DAM module is embedded before each bidirectional fusion node in BiFPN. This enables deformable alignment of cross-scale features followed by weighted fusion, forming the BiFPN-DAM network. In the motion prediction module, dynamic Kalman filtering is employed alongside a confidence-based observation noise modulation function to achieve adaptive updates. The main workflow of DAM-Track is illustrated in Fig. 7.

As shown in Fig. 7, the proposed object tracking algorithm adopts the ResNet-18-DAM network as the backbone feature extractor. The high-level features of this network dynamically focus on key deformation regions of the target through adaptive receptive fields. Subsequently, a bidirectional feature pyramid network enhanced by deformable attention is introduced for cross-scale feature fusion, leveraging bidirectional information flow and adaptive weighting mechanisms to improve the discriminative power of multi-scale representations. Finally, a dynamic Kalman filter models target motion and predicts its state, incorporating a confidence-driven observation fusion strategy. When the confidence of a detection bounding box exceeds the threshold, the observation value is directly adopted to halt error accumulation; otherwise, the predicted bounding box and the detection bounding box are fused to generate the final tracking output.

## Results and analysis

### Performance verification of ResNet-18-DAM feature extraction network

To validate the performance of the feature extraction network ResNet-18-DAM, the study designed ablation experiments to analyze the algorithm’s effectiveness. To ensure the reliability of the experiments, the same computer was used, and high-performance hardware and software were selected for testing. The experimental environment setup is detailed in Table 2.

**Table 2:** Experimental environment configuration.

/	Configuration item	Detailed information
Hardware part	CPU	Intel Xeon E52680 v4, 14 cores, 28 threads, 2.4 GHz clock frequency, dynamic acceleration frequency 5.3 GHz
	GPU	NVIDIA GeForce RTX 3080, 10GB GDDR6X video memory, core frequency 1440 MHz, maximum Rui frequency 1710 MHz
	RAM	64GB DDR4, 3200 MHz
	Storage	1 TB NVMe SSD, 2 TB SATA SSD
Software part	Operating system	Ubuntu 20.04 LTS, 64-bit
	Programming language	Python 3.8

**Fig. 8:**
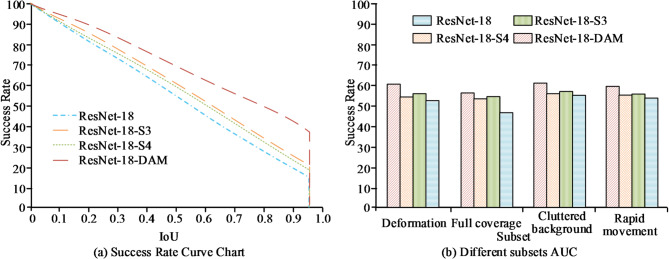
Success rate statistics of different networks on the LaSOT dataset (dataset source: https://cis.temple.edu/lasot/).

As shown in Table 2, the selected CPU and GPU devices provided high performance, and the storage capacity was sufficient to ensure normal operation of the algorithms. The study selected two authoritative single-object tracking datasets, LaSOT and GOT-10k, for ablation experiments to comprehensively evaluate the core capabilities of the feature extraction network. The LaSOT dataset comprises 1,400 long video sequences totaling over 3.5 million frames, with an average sequence duration of approximately 84 s. It is annotated with 14 challenging attributes, including deformation and full occlusion, specifically designed to evaluate trackers’ robustness and discriminative power under long-duration tracking and complex appearance changes. The GOT-10k dataset comprises over 10,000 video sequences, designed with the core principle of achieving zero overlap in object categories between training and testing sets. To systematically quantify the contribution of each module, a hierarchical and progressive ablation experiment was designed. The experiment first established a standard twin network tracking framework as a unified evaluation platform, ensuring that all other aspects-architecture, training data, and hyperparameters-remained identical except for the feature extraction network. Within this framework, the following networks were sequentially embedded: the original ResNet-18, ResNet-18-S3 (DAM embedded only in stage 3), ResNet-18-S4 (DAM embedded only in stage 4), and the complete ResNet-18-DAM network. This sequence validated the necessity of the DAM module for modeling geometric deformations. This design choice is based on the specialized roles of different network stages: Stage 3 features contain rich spatial details crucial for handling local deformations, while Stage 4 features possess stronger semantic abstraction and context awareness, making them more sensitive to the object’s overall pose and motion trends. This progressive combination allows for separate evaluation of DAM’s contributions. The experiment first statistically analyzes the success rates of different networks on the LaSOT dataset, with results shown in Fig. 8.

**Fig. 9:**
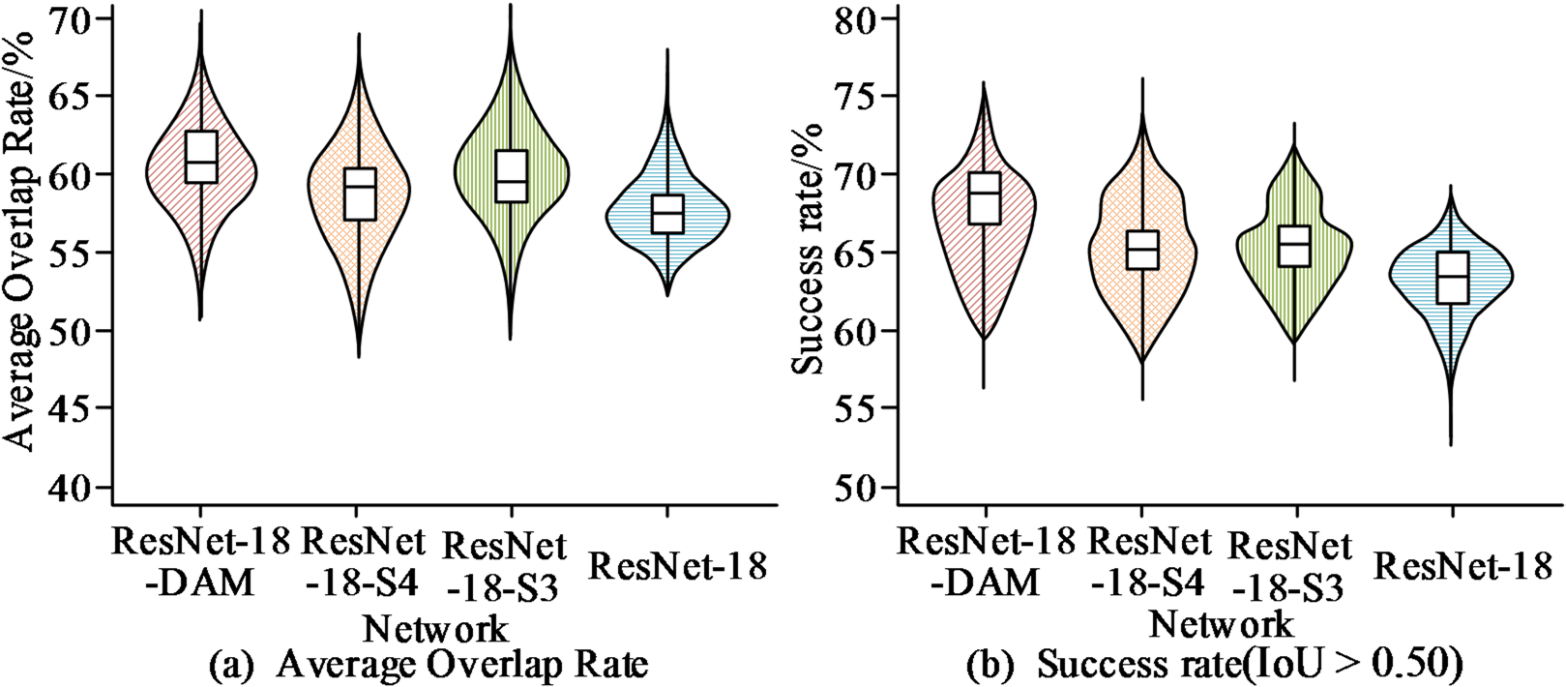
Comparison of average overlap rate and success rate of different networks on the GOT-10k test set.

As shown in Fig. 8a, as the Intersection over Union (IoU) threshold gradually increases from 0.0 to 1.0, the tracking success rates of all networks exhibit a monotonic decreasing trend. Among them, the complete ResNet-18-DAM network consistently maintains the highest performance level, with its curve remains significantly above the other three curves throughout. Particularly in the high-threshold range above 0.7, its decline rate is the most gradual. In contrast, variants embedding the attention module only in the third or fourth stage of the network show improvement over the original ResNet-18 baseline, but their curves remain noticeably lower than that of the complete model. From Fig. 8b, in evaluations across various challenge subsets of LaSOT, the ResNet-18-DAM network demonstrates significant advantages. On the two core challenges of “Deformation” and “Full coverage,” its success rates reach 60.3% and 56.4%, respectively, representing substantial improvements over baseline ResNet-18. This directly validates the effectiveness of the deformable attention module in focusing on key regions. Subsequently, the study tested the performance of different networks on the GOT-10k dataset to verify the generalization capability of the network. The statistical results of average overlap rate and success rate (IoU > 0.50) for different networks on the GOT-10k test set are shown in Fig. 9.

**Fig. 10:**
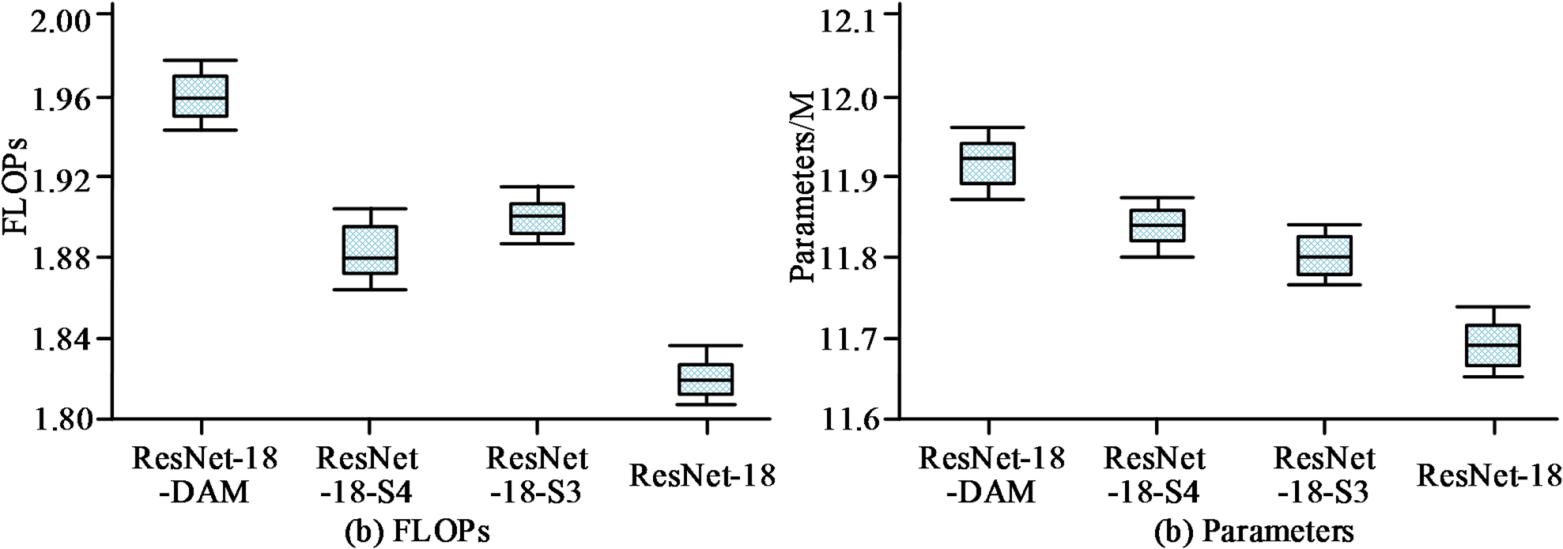
Comparison of computational efficiency among different feature extraction networkson the GOT-10k test set.

As shown in Fig. 9a, the original ResNet-18 network achieved an average overlap rate of 57.8%. The ResNet-18-S3 model performed slightly better, with an average overlap rate of 59.2%, while the proposed ResNet-18-DAM network attained the best performance, reaching an average overlap rate of 61.5%. From the success rate data in Fig. 9b, the original ResNet-18 network achieved only a 63.5% success rate. Variant networks incorporating the DAM attention mechanism showed improvements in success rates, with ResNet-18-S3 increasing to 65.8%. In comparison, the ResNet-18-DAM network achieved an even higher success rate of 68.4%. These experimental results confirm that collaboratively embedding the DAM attention mechanism in the third and fourth stages of the ResNet-18 network can effectively guide the network to learn more generalized visual representations. Subsequently, the study tested the computational efficiency of the feature extraction network, with evaluation metrics including Floating Point Operations (FLOPs) and parameter count. The specific experimental results are presented in Fig. 10.

As illustrated in Fig. 10a, the computational load of standard ResNet-18 is 1.82 GFLOPs. The FLOPs of variant networks embedding a single deformable attention module (ResNet-18-S3 and ResNet-18-S4) increase to approximately 1.90 GFLOPs and 1.88 GFLOPs, respectively. In contrast, the complete ResNet-18-DAM network has a computational load of approximately 1.96 GFLOPs, representing an overall increase of less than 8% compared to the baseline ResNet-18, thus maintaining favorable inference efficiency. From Fig. 10b, in terms of model size, the parameter count of the original ResNet-18 network is 11.69 M. The additional parameters introduced by the DAM module are minimal, with the complete model’s parameter count only increasing to 11.92 M. These data indicate that the proposed ResNet-18-DAM network achieves significant improvement in tracking performance with extremely low computational and storage overhead.

### Performance validation of the DAM-Track algorithm incorporating the DAM attention mechanism

After validating the effectiveness of the ResNet-18-DAM feature extraction network incorporating a deformable attention mechanism, the study further conducted tests on the highly challenging MOT 20 multi-object tracking benchmark dataset within the MOTChallenge evaluation platform to comprehensively assess the overall performance of the proposed object tracking algorithm, DAM-Track. This dataset comprises eight high-definition video sequences, featuring scenes concentrated in extremely crowded public spaces such as train stations and large gatherings. On average, each frame requires simultaneous tracking of up to 246 pedestrian targets. The resulting frequent and severe occlusions pose an extreme challenge to the target detection, identity separation, and long-term trajectory association capabilities of tracking algorithms. Following the platform’s principle of fair comparison, Common trackers including ByteTrack, Motion Transformer (MOTR), Global Context-Aware Tracker (GCNet), Observation-Centric SORT (OCSORT), and Simple Online and Realtime Tracking (DeepSORT) were selected as comparison models. OC-SORT), and Simple Online and Realtime Tracking (DeepSORT) as comparison algorithms. Evaluation metrics included Multiple Object Tracking Accuracy (MOTA), Multiple Object Tracking Precision (MOTP), Identity F1-score (IDF1), and Higher Order Tracking Accuracy (HOTA). The algorithms were tested for overall accuracy, identity retention, and balance. MOTA represents the tracking system’s comprehensive accuracy, with higher values indicating better overall performance. MOTP is the average positioning accuracy metric for successful target matching. Higher values indicate more precise bounding boxes. IDF1 is a key metric for measuring identity retention capability; a higher IDF1 indicates stronger algorithmic ability to distinguish different objects and maintain ID consistency. HOTA is a metric that comprehensively evaluates detection, association, and localization performance, obtained by calculating the average performance across different association thresholds. The experimental data obtained under the MOTChallenge platform standard protocol are shown in Table 3.

**Table 3:** Statistical table of MOTA, MOTP, IDF1, and HOTA for different tracking algorithms on the MOT 20 multi-object tracking benchmark dataset (dataset source: https://motchallenge.net/data/MOT20/).

Algorithm	MOTA/%	MOTP/%	IDF1/%	HOTA/%
ByteTrack	75.6	78.9	74.3	60.8
MOTR	72.4	77.5	67.6	56.8
GCNet	75.0	79.2	75.5	62.0
OC-SORT	78.2	79.5	76.0	63.4
DeepSORT	71.3	76.8	69.6	59.3
DAM-Track	77.5	80.4	77.0	63.3

As shown in Table 3, DAM-Track demonstrates outstanding performance on the MOT20 dataset, achieving MOTA, MOTP, IDF1, and HOTA values of 77.5%, 80.4%, 77.0%, and 63.3%, respectively. Among the four core metrics, MOTP reaches 80.4%, indicating that DAM-Track possesses the most precise localization capability. These experimental results collectively demonstrate the crucial role of the proposed ResNet-18-DAM feature extraction network and action filtering strategy in enhancing target discriminability and trajectory association accuracy.Finally, the study analyzes the performance of different tracking algorithms in terms of trajectory completeness, stability, and error composition. The evaluation metrics include the proportion of Mostly Tracked trajectories (MT), the proportion of Mostly Lost trajectories (ML), the number of Identity Switches (IDs), the count of False Positives (FP), and the count of False Negatives (FN). Among these, MT measures the tracker’s ability to achieve long-term successful tracking; a higher MT value indicates stronger continuous tracking capability. ML measures the extent of tracking failure; a lower value indicates fewer targets lost by the algorithm. IDs serve as a direct metric for assessing the degree of identity confusion; lower IDs indicate better identity continuity and more accurate data association. FP and FN represent the number of detection boxes mistakenly identified as targets and the number of real target boxes that were not detected or tracked, respectively. The experimental results are presented in Table 4.

**Table 4:** Statistical table of trajectory quality metrics for different tracking algorithmson the MOT 20 multi-object tracking benchmark dataset.

Algorithm	MT/%	ML/%	IDs	FP	FN
ByteTrack	51.2	14.2	1816	11,187	35,213
MOTR	48.7	16.0	1232	12,841	40,363
GCNet	50.3	14.6	1498	11,432	36,250
OC-SORT	52.9	13.3	1331	9928	31,567
DeepSORT	44.6	19.4	2875	13,238	40,469
DAM-Track	54.6	12.5	1035	10,285	32,960

As shown in Table 4, the tracking algorithm proposed in the study demonstrates excellent performance in trajectory quality. Its MT reaches 54.6%, the highest among all compared algorithms, while its ML is 12.5%, the lowest value, which intuitively reflects the algorithm’s exceptional ability to maintain trajectory stability during long-term tracking. In terms of identity consistency, the proposed algorithm achieves an IDs value of only 1,048, significantly lower than that of other algorithms. Regarding error composition, the FP and FN counts of the proposed algorithm are controlled at 10,285 and 32,960, respectively, both ranking among the best levels. These data collectively confirm that the proposed algorithm not only leads in comprehensive performance but also exhibits considerable advantages in trajectory continuity, stability, and identity consistency.

## Discussion and interpretation

To address the issues of low accuracy and insufficient anti-interference capability of existing object tracking algorithms in complex environments, this study embeds a Deformable Attention Module (DAM) into the ResNet-18 backbone network, enabling the network to dynamically adjust the receptive field and precisely focus on the key discriminative regions of the target. In the LaSOT long-term tracking benchmark test, the proposed method achieved success rates of 60.3% and 56.4% when dealing with target deformation and full occlusion challenges, respectively, significantly outperforming the baseline model. This performance improvement results from the DAM module’s ability to adapt the network to drastic appearance changes and learn more robust feature representations. This finding aligns with the research by Shao D et al., who integrated a spatial transformer network and a squeeze-and-excitation (SE) attention module into ResNet-18 to enhance facial expression recognition accuracy. Both studies validated the effectiveness of attention mechanisms in improving the feature discriminability of lightweight networks^32^. In the GOT-10k generalization capability test, the proposed network achieved the best performance with an average overlap rate of 61.5% and a success rate of 68.4%, demonstrating the excellent universality of the learned visual representations. This further indicates that the DAM module not only improves accuracy for specific tasks but also enhances the essential representational capacity of features. The research by Yang W et al., which integrated a Convolutional Block Attention Module (CBAM) into the ResNet-18 network to enhance key facial feature extraction, showed that the network can achieve a recognition accuracy of up to 89.55%^33^. These results collectively demonstrate that attention mechanisms can not only improve the accuracy of recognition tasks but also enhance the feature representation capability of models. More importantly, the performance advantages achieved in this study come at an extremely low computational cost. The introduction of the DAM module only increased the computational load to 1.96 GFLOPs and slightly raised the parameter count by 0.23 M to 11.92 M, reflecting an efficient design philosophy. This aligns with the research approach of Li J et al., who optimized algorithms to balance performance and complexity^34^. Additionally, the method by Xue C’s research team, which dynamically disables redundant layers in lightweight Vision Transformers to improve efficiency, shares the core concept of reducing computational resources with the dynamic attention mechanism proposed in this study^35^.

In addition, this study introduced BiFPN as the feature fusion network of the object tracking algorithm and optimized it with DAM. At the same time, the algorithm used dynamic Kalman filtering as the target prediction module to achieve continuous tracking. The experimental results indicate that the proposed object tracking algorithm achieves a MOTA of 77.5%, a MOTA of 80.4%, an IDF1 of 77.0%, and MT and ML of 54.6% and 12.5%, respectively, on the MOT20 dataset. These advantages mainly came from the DAM-optimized BiFPN and the dynamic Kalman filtering. The improved BiFPN used the dynamic attention mechanism and bidirectional feature fusion, which significantly enhanced the feature representation ability for multi-scale targets. The dynamic Kalman filtering effectively solved the occlusion problem through a noise adjustment mechanism adaptive to detection confidence. These results were consistent with the conclusions of previous studies but showed significant advantages in the complexity of application scenarios. Wang, H et al. replaced the original feature fusion network of Yolov4 with BiFPN to reduce computational consumption and embedded an attention mechanism in the feature extraction network. Their experiments showed that the improved object detection algorithm reduced the total number of parameters, increased the computational speed by 1.71 times, and improved the accuracy^36^. Wu, Q and his team also used BiFPN to replace the feature fusion network of Yolov5s and embedded an attention mechanism to improve the accuracy of object detection. Their comparison experiments showed that the improved model achieved an average accuracy of 94.8% and reduced the number of parameters by 30.72%^37^. However, these studies mainly focused on fixed targets or targets with simple movements and did not fully consider the applicability of the algorithm in complex dynamic environments. In comparison, this study systematically verified the applicability of BiFPN in complex dynamic tracking environments through a series of optimization designs, especially showing significant advantages in handling challenging scenarios such as target occlusion, fast motion, and scale variation.

## Conclusions and recommendations

This paper addressed key problems of low accuracy and weak anti-interference ability in existing object tracking algorithms under complex scenarios by employing ResNet-18 and a refined BiFPN as the core feature extraction and fusion networks, and introduced a DAM-based object tracking algorithm DAM-Track. The paper applies an improved dynamic Kalman filter to enhance the algorithm’s adaptability to target motion states. Experiments showed that ResNet-18 with DAM significantly enhanced the feature extraction capability of the network. Simultaneously, the improved BiFPN overcomes the performance limitations in feature fusion of traditional algorithms in scenes with target movement and occlusion. In addition, the improved dynamic Kalman filter effectively enhanced the algorithm’s adaptability to motion variations of the target. The algorithm proposed in the study, while demonstrating good performance on conventional visible light datasets, still requires further validation of its generalization ability in complex scenarios such as, night-time, aerial, and multi-camera. These scenarios often involve varying imaging quality and unconventional motion patterns, which can introduce bias into the confidence adjustment mechanism of the dynamicman filter. Therefore, future research also needs to establish a multimodal uncertainty propagation model to enhance the robustness of dynamic filtering under complex sensing conditions by quantifying the correlation between imaging and confidence.

## Data Availability

The datasets used and/or analysed during the current study available from the corresponding author on reasonable request.
